# Acid bone lysate activates TGFβ signalling in human oral fibroblasts

**DOI:** 10.1038/s41598-018-34418-3

**Published:** 2018-10-30

**Authors:** Franz Josef Strauss, Alexandra Stähli, Lucian Beer, Goran Mitulović, Valentina Gilmozzi, Nina Haspel, Gerhild Schwab, Reinhard Gruber

**Affiliations:** 1 Department of Oral Biology, Medical University of Vienna, Sensengasse 2a, 1090 Vienna, Austria; 2 Department of Conservative Dentistry, School of Dentistry, University of Chile, Sergio Livingstone 943, Santiago, Chile; 3 Department of Periodontology, School of Dental Medicine, University of Bern, Freiburgstrasse 7, 3010 Bern, Switzerland; 4 Department of Biomedical Imaging and Image-guided Therapy, Medical University Vienna, Währinger Gürtel 18-20, 1090 Vienna, Austria; 5 Christian Doppler Laboratory for Cardiac and Thoracic Diagnosis and Regeneration, Medical University Vienna, Währinger Gürtel 18-20, 1090 Vienna, Austria; 6 Clinical Department of Laboratory Medicine Proteomics Core Facility, Medical University Vienna, Währinger Gürtel 18-20, 1090 Vienna, Austria

## Abstract

Demineralized bone matrix is a widely used allograft from which not only the inorganic mineral but also embedded growth factors are removed by hydrochloric acid (HCl). The cellular response to the growth factors released during the preparation of demineralized bone matrix, however, has not been studied. Here we investigated the *in vitro* impact of acid bone lysate (ABL) prepared from porcine cortical bone chips on oral fibroblasts. Proteomic analysis of ABL revealed a large spectrum of bone-derived proteins including TGF-β1. Whole genome microarrays and RT-PCR together with the pharmacologic blocking of TGF-β receptor type I kinase with SB431542 showed that ABL activates the TGF-β target genes interleukin 11, proteoglycan 4, and NADPH oxidase 4. Interleukin 11 expression was confirmed at the protein level by ELISA. Immunofluorescence and Western blot showed the nuclear localization of Smad2/3 and increased phosphorylation of Smad3 with ABL, respectively. This effect was independent of whether ABL was prepared from mandible, calvaria or tibia. These results demonstrate that TGF-β is a major growth factor that is removed upon the preparation of demineralized bone matrix.

## Introduction

Bone grafts are regularly used for augmentation in implant dentistry, oral and maxillofacial surgery, besides other medical fields including orthopedics and traumatology dealing with bone reconstructions^1,2^. Freshly prepared bone autografts are considered gold standard in reconstructing large and complex bone defects due to the osteoconductive surface and the presence of osteogenic cells that can contribute to bone formation^3^. Furthermore, growth factors released upon autograft resorption are supposed to support bone regeneration, even though evidence to support this claim is poor. Similar to the resorption of autografts by osteoclasts, demineralization of allografts by hydrochloric acid does not only remove the mineral phase^4^. Hydrochloric acid also removes a fraction of growth factors intrinsic to bone. The biological activity of the respective acid bone lysate (ABL), which are discarded upon the preparation of demineralized bone matrix, has not been characterized so far.

Bone is a rich source of growth factors including TGF-β1^5,6^. Pioneer work of purification and characterization of TGF-β1 released by hydrochloric acid and other methods dates back to the 1980s^6–8^. With the introduction of proteomics, bone extraction protocols were refined^9^ still including demineralization of bone by hydrochloric acid^10^. The concentration of TGF-β1 with around 0.5 ng/ml in bone lysates is conserved among skeletal areas and gender^5^. *In vivo*, TGF-β1 is stored in a latent form and can be released and activated by osteoclasts^11–13^. TGF-β1 released during bone remodeling induces migration of mesenchymal stem cells^14,15^ and targets osteoclasts^16^. However, the activity of TGF-β1 and other growth factors in ABL has not been studied recently.

Bioassays with TGF-β-responsive cells are appropriate to determine TGF-β activity in ABL. We previously used oral fibroblasts to detect TGF-β1 activity in supernatants of freshly prepared bone chips^17^ and enamel matrix derivatives^18^. Moreover, the adsorption of TGF-β1 from these preparations to collagen matrices commonly used for guided bone regeneration was determined by gene expression changes of oral fibroblasts^19,20^. The selection of genes was based on proteomic analysis and a whole genome microarray resulting in a panel of TGF-β target genes including interleukin 11 (IL11), proteoglycan4 (PRG4), and NADPH oxidase 4 (NOX4)^21,22^. Further support for activation of TGF-β signaling comes from phosphorylation and translocation of Smad3 into the nucleus^23^.

Here we report the protein composition of ABL and determine the respective biological activity for oral fibroblasts. Based on a whole genome microarray and RT-PCR approach including the pharmacologic blocking of TGF-β receptor type I kinase with SB431542^24^, ABL caused a robust activation of TGF-β signaling. ABL also reduced the expression of osteogenic and adipogenic differentiation markers in calvaria cells and 3T3-L1 cells, respectively. Our findings suggest that TGF-β is among the major growth factors being discarded upon the demineralization of bone matrix.

## Results

### ABL contains a large spectrum of proteins including TGF-β

**Figure 1:**
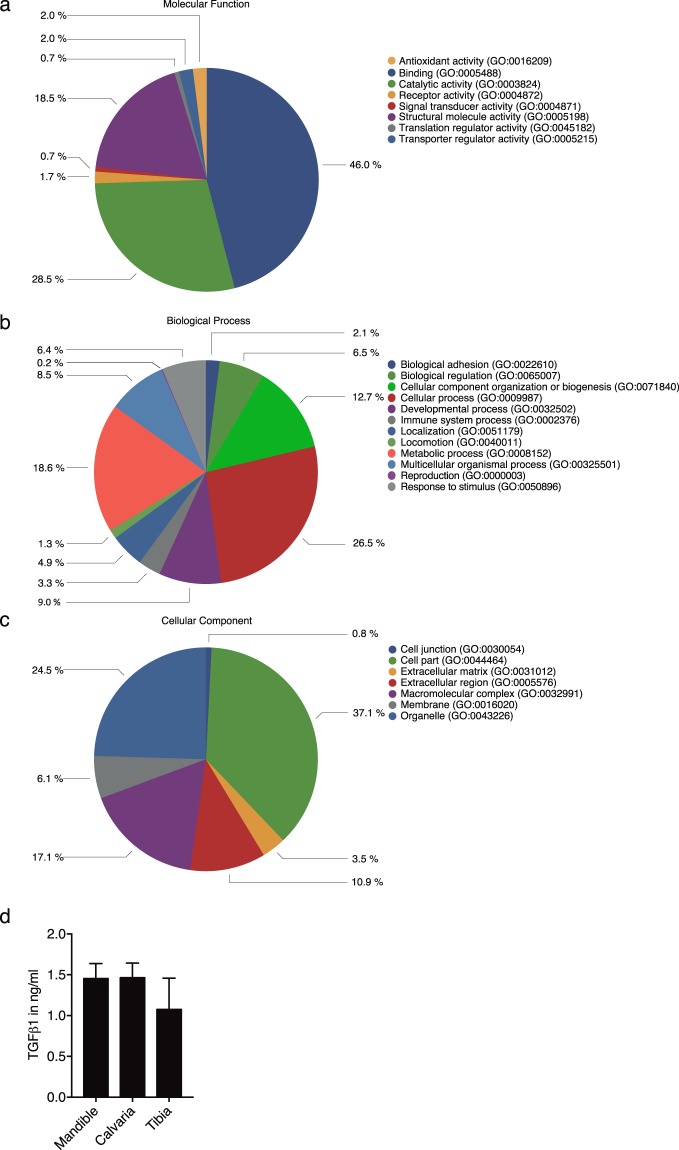
Acid bone lysate (ABL) contains a large spectrum of proteins including TGF-β. Panther analysis of molecular function (**a**) cellular component (**b**) and class (**c**) of proteins in ABL. (**d**) TGF*-*β1 immunoassay of different bone regions: mandible, calvaria and tibia. N = 3–5. Data represent the mean ± SD.

**Figure 2:**
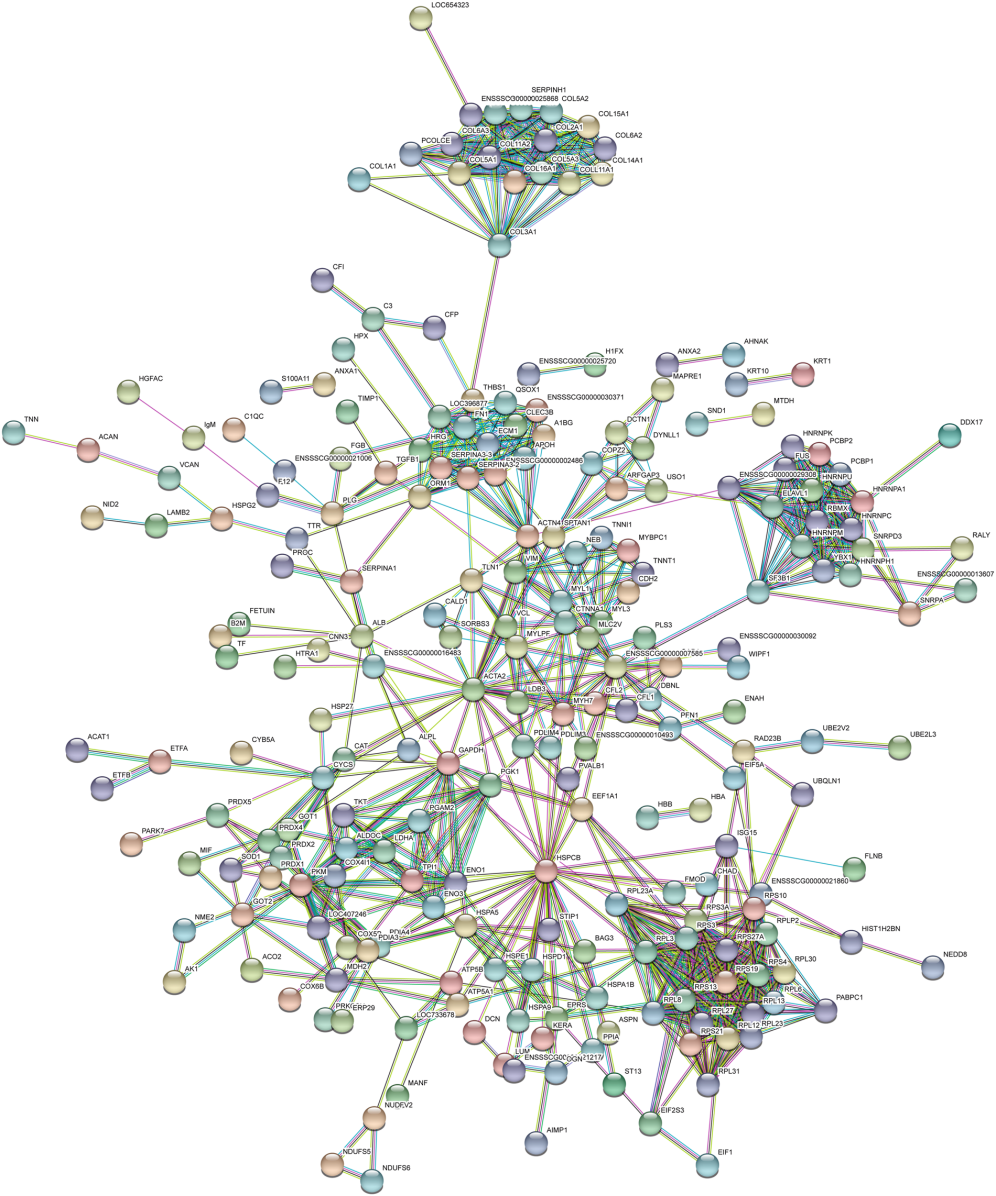
STRING representation of a network involving the proteins detected in ABL. Different line colors represent the types of evidence for the association between proteins.

To understand the complexity of ABL with respect to a possible cellular response, a proteomic analysis was performed. In support of previous proteomic analysis of bone-conditioned medium (BCM)^22^, we detected a wide range of proteins including growth factors with a possible impact on cellular function (Figs 1 and 2). Proteomic analysis of ABL revealed 394 proteins including TGF-β1 (Suppl. Table 1). Panther classified ABL proteins into 8, 12 and 7 groups according to their molecular function (Fig. 1a), biologic process (Fig. 1b) and cellular component (Fig. 1c), respectively. The majority of proteins were linked to transporter regulator activity (46%) and catalytic activity (28.5%) (Fig. 1a). Cellular process and metabolic process were represented by 26.5% and 18.6% of the proteins, respectively (Fig. 1b). The majority of the proteins originated from cells (37.1%) and organelles (24.5%) (Fig. 1c). The presence of active TGF-β1 in ABL was 1.3 ± 0.2 ng/ml independent of the bone source: mandible, calvaria or tibia (Fig. 1d). According to the STRING analysis tool, proteins in ABL revealed multiple clusters including ribosomal protein and collagens and also showed TGF-β1 (Fig. 2).

### ABL maintains cell viability

**Figure 3:**
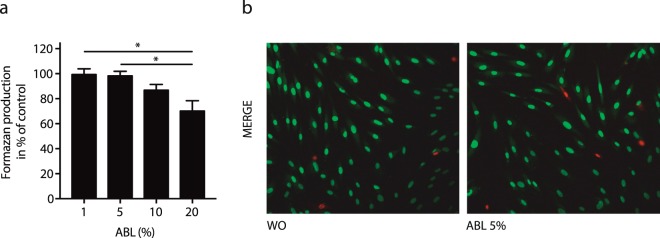
Viability of primary oral fibroblast exposed to acid bone lysate (ABL). Cell viability staining of primary oral fibroblast upon exposure to ABL was tested by MTT assay (**a**) and Live-Dead staining (**b**). The results from these experiments demonstrated that stimulation with ABL at 5% is highly biocompatible with oral fibroblast. Live-Dead staining was done with viable cells appearing in green and dead cells in red. N = 3–5. Data represent the mean ± SD relative to the control. *P < 0.05, by Kruskal-Wallis test with Dunn’s multiple comparisons correction.

To evaluate the impact of ABL on cell viability, the formation of formazan was determined. Dose-response curves indicate that a concentration of 5% ABL is appropriate to maintain viability of human gingival fibroblasts (Fig. 3a). Cell viability at 5% acidy bone lysate was further confirmed by live-dead staining (Fig. 3b). Thus, 5% of acid bone lysate was selected to study the whole genome response in gingival fibroblasts.

### ABL provokes changes in gene expression based on a whole genome assay

**Figure 4:**
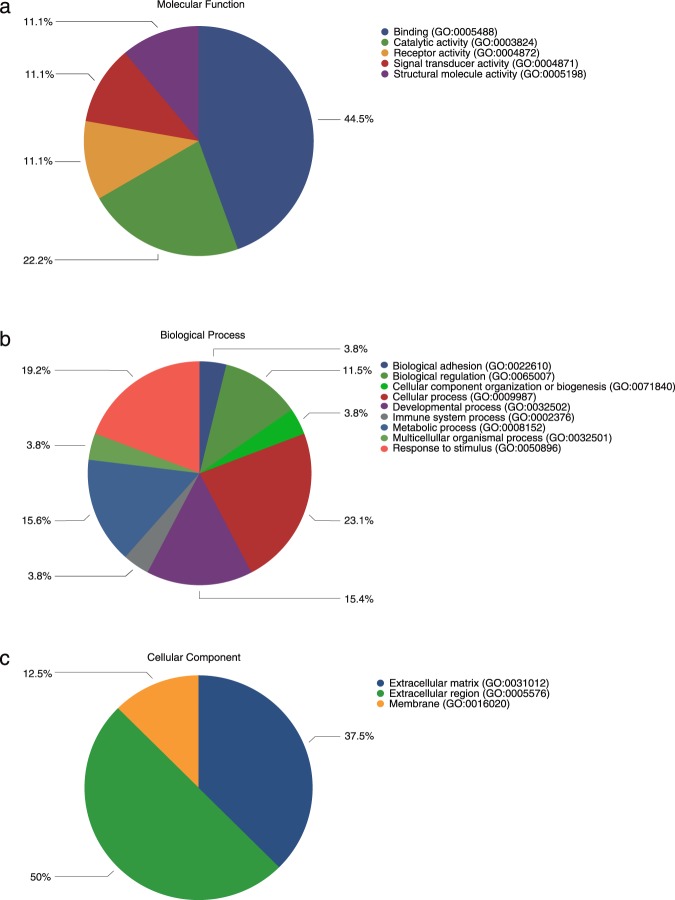
Acid bone lysate (ABL) provokes changes in gene expression based on gene arrays. Eighteen genes were at least 10-fold up- and down-regulated by ABL (Table 2). Panther analysis of molecular function (**a**) biological process (**b**) and cellular component (**c**) of most regulated genes in oral fibroblasts by ABL.

To determine the overall cellular response of oral fibroblasts to ABL, a whole genome gene assay was performed. Overall, 1527 genes are changed ≥ 1.5-fold (Suppl. Table 2). Among those genes, 17 genes (IL11, NOX4, PRG4, IL33, MMP10, ADAMTS5, MMP13, BMP2, COMP, INHBA, AREG, COL10A1, CXCL5, PMEPA1, TSPAN13, GPR183, ESM1) and 1 gene (PTX3) are at least 10-fold up- and down-regulated, respectively (Table 2). To gain information on the significantly expressed genes, we functionally categorized them using the WEB-based Gene Stet Analysis Toolkit (WebGestalt) database. GO-term analysis revealed that up-regulated genes in response to ABL were associated with the TGF-β signaling pathway (adj. P = 0.000045; enrichment score 12.12; genes: TGF-β1; BMP2, BMP6, ID3, ID1, INHBA, COMP) besides other signaling pathways. Panther classified the most regulated genes into 5, 9 and 3 groups according to their molecular function (Fig. 4a), biologic process (Fig. 4b) and cellular component (Fig. 4c), respectively. The majority of genes were linked to binding (44.5%) and catalytic activity (22.2%) (Fig. 4a). Cellular process and response to stimulus were represented by 23.1% and 19.2% of the genes, respectively (Fig. 4b). The majority of the genes were related to the extracellular region (50.0%) and extracellular matrix (37.5%) (Fig. 4c).

**Table 2:** Genes with at least 10x changes in oral fibroblasts exposed to ABL.

Gene Symbol	Regulation	Fold Change
IL11	up	85.5
IL33	up	30.3
MMP10	up	24.5
NOX4	up	20.5
ADAMTS5	up	20.4
MMP13	up	19.2
PRG4	up	17.0
BMP2	up	16.2
COMP	up	14.1
INHBA	up	14.0
AREG	up	13.5
COL10A1	up	13.3
CXCL5	up	12.7
PMEPA1	up	12.5
TSPAN13	up	12.5
GPR183	up	12.0
ESM1	up	10.3
PTX3	down	16.6

### ABL incites changes in gene expression via activation of TGF-β signalling

**Figure 5:**
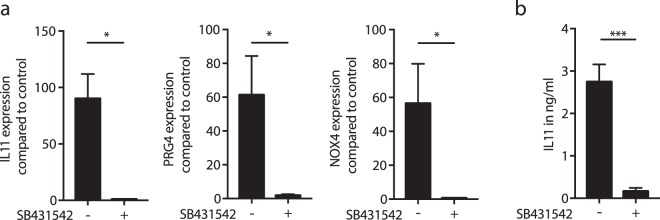
Acid bone lysate (ABL) changes the expression of selected genes via TGF-β signaling. Addition of TGF- β receptor 1 kinase antagonist SB431542 to ABL blocks the expression of the selected genes (**a**). Immunoassay of IL11 supports the previous findings at the protein level (**b**). N = 3–5. Data represent the mean ± SD. *P < 0.05, ***P < 0.001, by two-tailed Mann-Whitney test.

**Figure 6:**
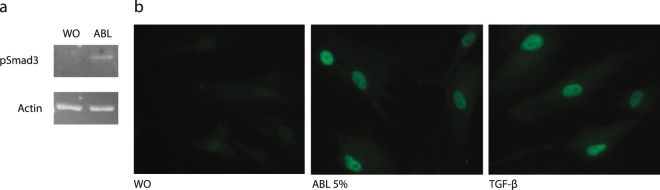
Acid bone lysate (ABL) activates TGF-β-Smad2/3 signaling in primary oral fibroblasts. Incubation of gingival fibroblasts with ABL also caused an increased phosphorylation of Smad3 (**a**). Representative immunofluorescence confirmed the translocation of Smad2/3 into the nucleus upon stimulation with ABL (**b**). Treatment with 10 ng/ml of TGF-β was used as a positive control.

In a pilot experiment with the gene array (n = 1), SB431542 blocked the expression of all 17 highly up-regulated genes to less than 2-fold, except for ESM1 (3.3-fold) and CXCL5 (5.1-fold) suggesting that the TGF-β receptor I kinase mediates the respective effect of ABL (Suppl. Table 3). In support of the gene array, RT-PCR and immunoassay confirmed the increased expression of IL11, PRG4, and NOX4 by ABL and also their suppression by SB431542 (Fig. 5a). At protein level, ABL increased IL11 release into the cell-culture supernatant and these effects were blocked by SB431542 (Fig. 5b). Further proof for activation of TGF-β signalling comes from gingival fibroblasts exposed to ABL showing increased phosphorylation of Smad3 (Fig. 6a) and an accumulation of Smad2/3 in the nucleus (Fig. 6b).

### ABL from various sources supports TGF-β signalling

**Figure 7:**
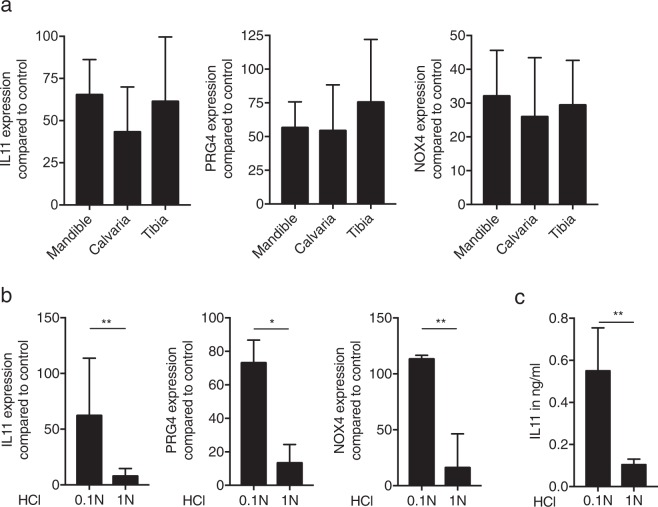
Acid bone lysate (ABL) obtained from mandible, calvaria and tibia produces an equivalent gene expression and TGF-β activity is highly dependent on hydrochloric acid concentration. ABL obtained from different sources (mandible, calvaria and tibia) produces a similar response on selected genes (**a**). ABL prepared with 0.1 N hydrochloric acid induces a higher TGF-β activity at mRNA level (**b**). Immunoassay of IL11 confirmed the higher activity of TGF-β at protein level with 0.1 N ABL (**c**). N = 3–5. Data represent the mean ± SD. P > 0.05, by Kruskal-Wallis test with Dunn’s multiple comparisons correction, *P < 0.05, **P < 0.01, by two-tailed Mann-Whitney test.

To understand if the embryologic origin of bone affects the activity of the respective ABL, bone chips were prepared from the mandible, the calvaria and the tibia^25^. All three independent preparations stimulated the expression of IL11, PRG4 and NOX4 in oral fibroblasts (Fig. 7a). Moreover, ABL prepared by 0.1 N HCl was most effective compared to lysates obtained with 0.01 N (data not shown), 1.0 N HCl (Fig. 7b) and also to lysates prepared with sodium tartrate buffer at pH 4.7 (Suppl. Fig. 2). In support of the RT-PCR, immunoassay confirmed the higher amount of IL11 in lysates obtained with 0.1 N (Fig. 7c).

### ABL decreases osteogenic and adipogenic differentiation markers

**Figure 8:**
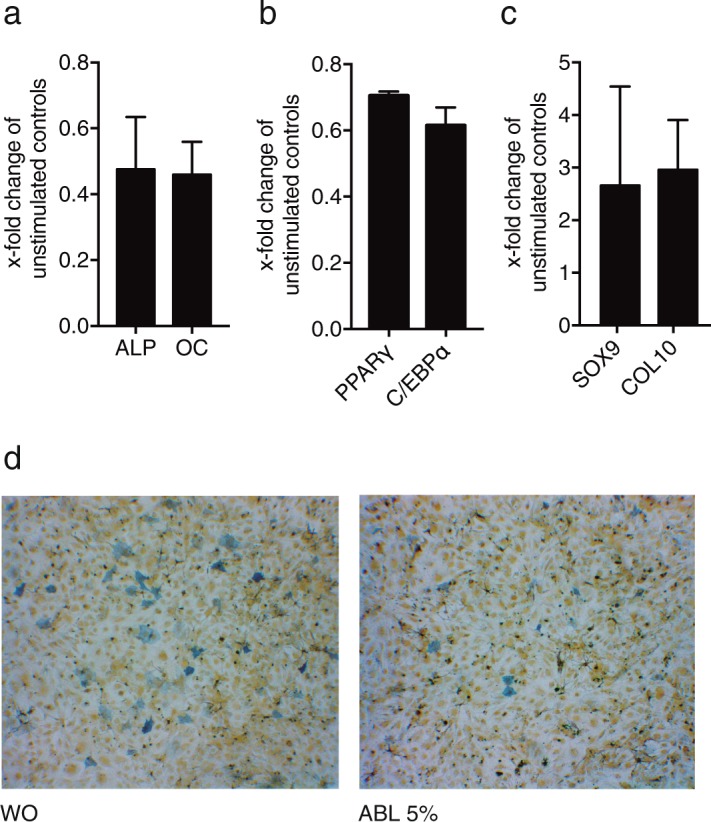
Acid bone lysate (ABL) decreases osteogenic and adipogenic differentiation. Exposure of osteogenic calvaria cells and adipogenic 3T3-L1 cells to ABL caused a considerable decrease of the marker genes alkaline phosphatase (ALP) and osteocalcin (OC) (**a**), as well as PPARγ and C/EBP (**b**), respectively. Chondrogenic ATDC5 cells showed a moderate increase of SOX9 and COL10 (**c**). Histochemical staining of alkaline phosphatase activity confirmed the findings from gene expression in osteogenic calvaria cells (**d**). N = 3–5. Data represent the mean ± SD.

In agreement with our previous work^17^, exposure of osteogenic calvaria cells and adipogenic 3T3-L1 cells to ABL caused a considerable decrease of the marker genes alkaline phosphatase (ALP) and osteocalcin (OC) (Fig. 8a), as well as PPARγ and C/EBP (Fig. 8b), respectively. Chondrogenic ATDC5 cells showed a moderate increase of SOX9 and COL10A (Fig. 8c). Histochemical staining of alkaline phosphatase activity confirmed the findings from gene expression (Fig. 8d).

## Discussion

Bone is a rich source of growth factors that are released by osteoclasts during remodeling^14,15^, fracture healing^26^, but also during the preparation of demineralized bone matrix. Previous immunoassays^5–8^ and today’s proteomic analysis^9,10^ revealed the composition of growth factors and other molecules stored within the bone matrix. If, however, the respective growth factors released by HCl can induce a cellular response remained unclear. The study described herein revealed that, although ABL contains a large spectrum of proteins including growth factors, all 17 genes most strongly up-regulated by ABL in oral fibroblasts, including IL11, NOX4 and PRG4, required activation of the TGF-β receptor type I kinase. In support of TGF-β receptor type I kinase signalling, ABL activated phosphorylation and nuclear accumulation of Smad3. Thus, demineralization by HCl caused the release of TGF-β into the acid bone lysate that is biologically active based on a panel of *in vitro* assays.

Our findings confirm previous observations that bone is a rich source of TGF-β1^5–8^, with a concentration of around 0.5 ng/ml in bone lysates of different skeletal areas^5^. Moreover, TGF-β being stored in the bone matrix in its latent form can be activated by low pH^27^. Also in line with our findings is that TGF-β of demineralized bone matrix maintains its activity^28^. In vital bone, TGF-β1 released by osteoclasts during bone remodeling controls migration of mesenchymal stem cells^14,15^ and also acts on osteoclasts^16^. Our pioneering research shows that TGF-β1 released during the preparation of demineralized bone matrix caused a major increase of TGF-β target genes, including IL11, NOX4 and PRG4. Support for this conclusion comes from our findings that in the presence of a TGF-β receptor I kinase inhibitor, ABL failed to change gene expression. These data are in support of previous research on BCM^21^ and enamel matrix derivative^19^ which also induces the expression of IL11, NOX4 and PRG4 mediated by TGF-β receptor type I kinase activity. Further confirmation for the activation of TGF-β signaling, comes from our observations that ABL increased phosphorylation and nuclear accumulation of Smad2/3, similar to research with recombinant TGF-β1^29^. Taken together, ABL holds a TGF-β activity as indicated by our bioassays with oral fibroblasts.

As a consequence of the TGF-β activity, based on our gene array approach, IL11, NOX4 and PRG4 were highly increased in oral fibroblasts by ABL. Considering that TGF-β activity is removed from bone grafts during demineralization, the question arises whether this change in gene expression has a biological or even clinical relevance. Clearly this is speculation but nevertheless these genes are involved in bone regeneration and wound healing. IL11 is a member of the IL6 family of cytokines and has been regarded as a target gene to investigate down-stream TGF-β signalling pathways in lung fibroblasts^30^, periodontal ligament and gingival fibroblasts^31^ and together with BMP-2, can accelerate bone regeneration^32^. NOX4 generates intracellular superoxide and also modulates osteoblasts BMP-2 activity^33^. Hydrogen peroxide^34^ reduces osteoblast differentiation and expression of alkaline phosphatase^35^ but it remains unclear if this mechanism explains our observations that ABL lowered osteogenic differentiation. PRG4 is expressed in the superficial zone of articular cartilage^36^ and supports endochondral bone formation^37^. Thus, considering that HCl lowers TGF-β from bone and that TGF-β target genes play a role in bone regeneration and remodeling, allografts likely possess a diminished capacity to use TGF-β signalling for graft consolidation. Moreover, whether the ABL-mediated increase of IL11, NOX4 and PRG4 expression has an impact on bone regeneration needs to be determined in appropriate animal models.

The clinical relevance of the present findings, therefore, may be related to the difference between bone autografts and allografts. While autografts are rich in TGF-β and other growth factors being stored in the bone matrix, demineralized bone matrix presumably contains only remnants of active TGF-β^38^. Moreover, HCl can deactivate pH sensitive growth factors in the demineralized bone matrix^39^. So overall, allografts are left with the growth factors that remain in the bone matrix while autografts that are rich in growth factors being liberated by osteoclasts. Can our observation now explain possible differences in the clinical behavior of autografts and allografts? On the one hand, TGF-β1 released during bone remodeling induces migration of mesenchymal stem cells^14,15^ and targets osteoclasts^16^, and exogenous TGF-β supports bone regeneration in the dog humerus^40^. On the other hand, however, TGF-β inhibited bone formation in rat bone chambers^41^ and mandibular defects^42^. TGF-β signalling also drives scar formation during wound healing^43^. In support for the latter concept are our findings that ABL considerably reduced osteogenic differentiation of calvaria cells, similar to TGF-β^44^ and bone BCM^17^ in MC3T3E1. Moreover, BCM loaded onto a collagen membrane can reduce bone formation in rat calvaria defects^45^. Thus, the clinical relevance of the present data has to be interpreted with caution, particularly with respect to the role of TGF-β1 released from bone grafts during graft consolidation. It is also not known if it is the content of TGF-β that explains the differential healing capacity of autografts and allografts *in vivo*.

Many questions remain to be answered. For example, whether ABL reflects the growth factors released by osteoclasts during resorption of autografts. Osteoclasts work at around pH 4 and can liberate growth factors by demineralization and their protease activity^46^. Our results showed that ABL prepared with a tartrate buffer at pH 4.7 moderately increased the expression of IL11 and NOX4 but not of PRG4, in contrast to the robust activation of TGF-β target genes obtained with ABL prepared with 0.1 M HCl. Osteoclasts might however activate latent TGF-β by proteases not being simulated in our experiment^11,47^. Considering that bone chips release TGF-β activity even at a neutral pH^17^, it remains unclear if the tartrate buffer liberates growth factors by decalcification of the bone matrix. Therefore, the data have to be interpreted with caution. Furthermore, it would be interesting to understand the response of cells other than those of the mesenchymal lineage including macrophages^48^ and endothelial cells^49^ that play a role during bone regeneration and thus graft consolidation. Our proteomic analysis, besides the work of others^10^, revealed a large spectrum of proteins that are released into ABL. Even though the most strongly regulated genes in oral fibroblasts are the consequence of TGF-β signalling, the possible cellular response to other growth factors and bioactive molecules within the ABL remain to be discovered. Another interesting approach would be to perform bone transplantation using mouse models with an osteoblast-specific TGF-β knock out^50^. This research can provide the scientific basis to refine protocols aiming to maintain growth factors and their activity during the preparation of allografts.

In conclusion, TGF-β is the major growth factor removed during the preparation of demineralized bone matrix that caused a robust activation of the respective signaling pathway in oral fibroblasts. These findings might provide a possible explanation to distinguish the performance of autografts and allografts at sites of bone augmentation.

## Methods

### Acid bone lysate

Bone was obtained from adult pigs within 6 h post-mortem (Fleischerei Leopold Hödl, Vienna, Austria). Bone chips were harvested from the mandible, calvaria, and tibia with a bone scraper (Hu-Friedy, Rotterdam, the Netherlands). Bone chips were washed with Dulbecco’s modified Eagle medium (DMEM) supplemented with antibiotics (Invitrogen Corporation, Carlsbad, CA, USA). Five grams of wet bone chips were incubated while being stirred with 50 ml of 0.01, 0.1 and 1.0 N HCl (10% weight/volume) or alternatively with 10 mM sodium tartrate buffer (pH 4.7) at room temperature. ABL was harvested after 16 h, centrifuged, and pH neutralized. After another centrifugation, ABL was filtered sterile and kept frozen at −20 °C. The stocks were thawed immediately before each experiment.

### Cell culture

Human gingiva was harvested from extracted wisdom teeth from patients who had given informed and written consent. An approval was obtained from the Ethics Committee of the Medical University of Vienna (EK NR 631/2007), Vienna, Austria. All experiments were performed in accordance with relevant guidelines and regulations. A total of three strains of fibroblasts were established by explant cultures and fewer than 10 passages were used for the experiments. Calvaria-derived osteoblasts were obtained according to a standard protocol previously described^51^. Briefly, mouse pups less than 5 days old were euthanized and their calvaria collagenase digested through a series of sequential digestions. The first 2 digests were discarded, and the subsequent digests were pooled and plated. The 3T3-L1 cell line was obtained from Christian Wolfrum (ETH Zürich, Zürich, Switzerland). Cells were seeded at a concentration of 30,000 cells/cm² onto culture dishes one day prior to stimulation. If not otherwise indicated, cells were exposed to 5% ABL in serum-free medium for 16 h. The inhibitor for the TGF-βRI kinase, SB431542 (Calbiochem, Merck, Billerica, MA, USA) was used at 10 µM.

### Cell differentiation

For osteogenic differentiation, calvaria cells were incubated in growth medium containing 50 μg/mL ascorbic acid and 10 mM beta glycerophosphate. For adipogenic differentiation, 3T3-L1 cells were incubated in growth medium containing 0.5 mM 1-methyl-3-isobutylxanthine (Sigma, St. Louis, MO, USA), 1 μM dexamethasone (Sigma) and 1 μg/mL insulin (Calbiochem, Merck Millipore), 10 μM indomethacin (Sigma), and 10 μM rosiglitazone (Sigma)^52^. Alkaline phosphatase staining was performed after 3 days. For histochemical staining of alkaline phosphatase, cells were fixed as indicated and incubated with a substrate solution containing naphthol AS-TR phosphate and fast blue BB salt (Sigma)^53^. After rinsing with distilled water, cultures were photographed.

### Cell viability

For viability experiments, gingival fibroblasts were incubated overnight with ABL at the indicated concentrations. MTT (3-[4,5-dimethythiazol-2-yl]-2,5-diphenyltetrazolium bromide; Sigma) solution at a final concentration of 0.5 mg/ml was added to each well of a microtiter plate (CytoOne) for 2 h at 37 °C. The medium was removed and formazan crystals were solubilized with dimethyl sulfoxide. Optical density was measured at 570 nm. Data were expressed as percentage of optical density in the treatment groups normalized to unstimulated control values. In addition, cell viability was assessed by Live-Dead staining kit from Enzo Life Sciences AG (Lausen, Switzerland).

### Mass spectrometry

Extracted proteins were precipitated using methanol/dichloromethane and digested with trypsin as described earlier^54^ (For detail see Suppl. Methods). Briefly, precipitated proteins were dissolved in 50 mM triethylammonium bicarbonate, and protein concentration was determined using the DeNovix DS-11 Microvolume Spectrophotometer (Wilmington, USA). Proteins were digested overnight at 37 °C using a trypsin/protein ratio of 1:50. Peptides were separated on a C18 µPAC (µ-Pillar-Arrayed-Column, PharmaFluidics, Gent, Belgium) using a nano RSLC UltiMate3000 (ThermoScientific, Vienna, Austria) separation system and detected with a Q-Exactive Plus Biopharma mass spectrometer.

A user defined injection program was used for sample injection and additional injector and trap column wash. Every sample injection was followed by two blank runs with injections of 2,2,2-trifluoroethanol for removal of possible samples remaining in the injector or on the trap column and prevention of carryover in the separation system. All database searches were performed using the in-house Mascot 2.6 and the most recent version of the Sus scrofa SwissProt database. All search results were refined and researched using Scaffold 4.6.5 (Proteome Software, Portland, OR). For the classification of the proteins the Panther system Version 13.1 (http://pantherdb.org) was used^55^. To predict protein-protein interactions String database was used^56^ (https://string-db.org). The mass spectrometry proteomics data have been deposited to the ProteomeXchange Consortium via the PRIDE^57^ partner repository with the dataset identifier PXD010145 and 10.6019/PXD010145”.

### Microarray analysis

Total RNA was harvested with the RNA Isolation Kit (Extractme, BLIRT S.A., Gdańsk, Poland). RNA quality was determined using the Agilent 2100 Bioanalyzer (Agilent Technologies, Santa Clara, CA, USA). Microarray analysis was performed using the SurePrint G3 Human Gene Expression v2 Microarray (Agilent Technologies, Santa Clara, CA, USA). Array image acquisition was performed with the Agilent G2505B Microarray Scanner and Feature Extraction software version 9.5 (Agilent). Background-corrected fluorescence intensity values were imported into GeneSpring v.15, log2-transformed, and then normalized by quantile normalization. A filtering step was applied in order to reduce the number of multiple hypotheses. Only genes for which at least 100% of the values in one of the two evaluated conditions were above the 60th percentile were used for further analysis. Differentially expressed mRNAs were identified by paired t-tests in GeneSpring. The resulting p-values were corrected for multiplicity by applying Benjamini-Hochberg adjustment to all p-values calculated for a time point with a false discovery rate (FDR) < 5%^58^. Genes with an adjusted p-value < 0.05 were considered significant.

### RT-PCR and immunoassay

Reverse transcription (RT) was performed with the SensiFAST™ cDNA Synthesis Kit (Bioline Reagents Ltd., London, UK). RT-PCR was done with SensiFAST™ SYBR® Kit using manufacturer’s instructions (Bioline). Amplification was performed with the StepOnePlus Real-Time PCR System (Applied Biosystems, Life Technologies, Carlsbad, CA, USA). Primer sequences are given in Table 1. Relative gene expression was calculated with the delta delta CT method. Reactions were run in duplicates. The supernatant was analyzed for IL11 and TGF-β1 using an immunoassay assay according to the manufacturer’s instructions (R&D Systems, Minneapolis, MN, USA).

**Table 1:** Primer sequences.

	Sequence_F	Sequence_R
hGAPDH	aag cca cat cgc tca gac ac	gcc caa tac gac caa atc c
hPRG4	cag ttg cag gtg gca tct c	tcg tga ttc agc aag ttt cat c
hNOX4	tct tgg ctt acc tcc gag ga	ctc ctg gtt ctc ctg ctt gg
hIL11	gga cag gga agg gtt aaa gg	gct cag cac gac cag gac
mbactin	cta agg cca acc gtg aaa ag	acc aga ggc ata cag gga ca
mC/EBP	caa gag ccg aga taa agc caa aca	gtg tcc agt tca cgg ctc ag
mCol10	gca tct ccc agc acc aga	cca tga acc agg gtc aag aa
mOC	ctg acc tca cag atg cca ag	gta gcg ccg gag tct gtt c
mPPARg	atc atc tac acg atg ctg gcc	ctc cct ggt cat gaa tcc ttg
mSox9	cag caa gac tct ggg caa g	tcc acg aag ggt ctc ttc tc
mALP	aac cca gac aca agc att cc	gag aca ttt tcc cgt tca cc

### Western blot

Cell extracts containing SDS buffer and protease inhibitors (PhosSTOP with cOmplete; Sigma, St. Louis, MO, USA) were separated by SDS-PAGE and transferred onto nitrocellulose membranes (Whatman, GE Healthcare, General Electric Company, Fairfield, CT, USA). Membranes were blocked and the binding of the first antibody (rabbit anti-pSmad3 Ser423/425, 1:500, Abcam, ab52903, Cambridge, UK), and actin (Santa Cruz Biotechnology, SCBT, Santa Cruz, CA, USA) was detected with the appropriate secondary antibody directly labeled with near-infrared dyes (LI-COR Biosciences, Lincoln, NE, USA) and visualized with the appropriate imaging system (LI-COR Biosciences). Acquired images were not processed.

### Immunofluorescence

Immunofluorescent analysis was performed on human gingival fibroblasts plated onto Millicell® EZ slides (Merck KGaA, Darmstadt, Germany) treated with ABL 5% for 30 min. Cells were fixed in paraformaldehyde and blocked in 1% BSA and 0.3% Triton in PBS at room temperature for 1 hour. Cells were subsequently incubated with Smad2/3 antibody (1:800, D7G7 XP® Rabbit mAb #8685, Cell Signaling, MA, USA) overnight at 4 °C. Alexa Fluor 488 secondary antibody (1:500; Anti-Rabbit, Cell signaling Technology, USA) was applied for 1 hour. Cells were washed and mounted onto glass slides. Fluorescent images were captured at 40x in oil immersion using a Zeiss Axiovert 200 M fluorescent microscope.

### Statistical analysis

All experiments were repeated three to five times. Bars show the mean and standard deviation of the cumulative data from all experiments. Statistical analysis was based on Mann-Whitney *U* test and Kruskal-Wallis test with Dunn’s multiple comparisons correction. Analyses were performed using Prism v7 (GraphPad Software, La Jolla, CA, USA). Significance was set at p < 0.05.

## Electronic supplementary material

Supplemental Information

Supplemental Table 1

Supplemental Table 2

Supplemental Table 3

## Data Availability

The datasets generated during and/or analyzed during the current study are available from the corresponding author on reasonable request.
