# Supplementary material for: Acid bone lysate activates TGFβ signalling in human oral fibroblasts
Source: Sci Rep. 2018 Oct 30;8:16065. doi: 10.1038/s41598-018-34418-3 (PMC6207660; doi:10.1038/s41598-018-34418-3)
Supplement: Supplementary file 1 — Supplemental Information [file 41598_2018_34418_MOESM1_ESM.pdf]

Supplementary Information

Acid bone lysate activates TGFβ signalling in human oral fibroblasts

Franz Josef Strauss, Alexandra Stähli, Lucian Beer, Goran Mitulović, Valentina Gilmozzi, Nina Haspel, Gerhild Schwab, Reinhard Gruber*

Supplemental Methods. Mass spectrometry.
Supplemental Table 1. Detailed information about all identified proteins.
Supplemental Table 2. List of the 1527 genes modulated by ABL in gingival fibroblasts (GF).
Supplemental Table 3. Selective inhibition of endogenous TGF-β1 signaling with SB431542 blocks the up-regulation of several ABL target genes in GF.
Supplementary Figure 1. Full-length blots, incubation of gingival fibroblasts with ABL caused an increased phosphorylation of Smad3.
Supplementary Figure 2. ABL prepared with tartrate-buffer at pH 4.7 changes the expression of IL11 and NOX4 but not PRG4.

Supplemental Methods

Mass spectrometry

Extracted proteins were precipitated using methanol/dichloromethane and digested with trypsin as described earlier (Fichtenbaum et al., 2016). Briefly, following acidic hydrolysis, proteins were dissolved in 50mM TEAB digestion buffer, pH 8.5 and were precipitated again using the modified Wessel-Fluegge method. Protein concentration was determined using the DeNovix DS-11 FX Spectrophotometer (Wilmington, USA) and were reduced using 5mM DTT (Dithiothreitol, Iodoaceticamide, Sigma-Aldrich, Vienna, Austria) for 30 minutes at 60°C, and alkylated for 30 minutes with 15mM IAA (Iodoaceticamide, Sigma-Aldrich, Vienna, Austria) in the dark. Finally, porcine trypsin (Promega, Vienna, Austria) was added in a ratio 1:50 (w/w). After 16 hours of incubation at 37°C, aliquots of 20µl were prepared and stored in 0.5ml protein low-bind vials (Eppendorf, Vienna, Austria) at -20°C until injection on next day. The nano HPLC Separation was performed using a nanoRSLC UltiMate 3000 HPLC system by Thermo Fisher. Mobile phases applied for sample loading, desalting, and separation were 0.01% aqueous heptafluorobutyric acid (HFBA) solution was used for sample loading applying a User Defined Program for sample injection. The loading mobile phase was delivered to the trap column at 30µl/min using the loading pump.

Mobile phases for peptide separation on the nano separation column were:

- A: 95% Acetonitrile (AcN), 5% Water, 0.1% Formic acid
- B: 50% AcN, 30% Methanol (MeOH), 10% 2,2,2-Trifluoroethanol (TFE), 10% Water, 0.1% Formic acid (FA)
- Autosampler loading solvent for sample injection was 0.1% aqueous TFA
- Wash of the injection needle, sample injection valve, and the trap column was performed using 100% TFE.

Trapping column used for sample loading, concentration and clean-up was a C18 PepMap, 3µm particle size, 300µm internal diameter and 5 mm length (ThermoFisher, Vienna, Austria). Nano chromatographic separation of peptides was performed on a C18 µPAC (µ-Pillar-Arrayed-Column, PharmaFluidics, Gent, Belgium). The pillars had an interpillar distance of 2µm, and the total separation path was 2µm. Both, the trap and the separation column were operated in a column oven at 45°C. Sample was loaded onto the trap column for 10 minutes when the valve switched the position and the nano gradient was directed through the trap column and onto the separation column. The trap column was switched back into the flow path of the loading column at 170 minutes in the runtime for equilibration and preparation of the following injection.

Separation was performed at 600nl/min and the gradient was formed as follows: An isocratic start with 2% B was maintained for 10 minutes and was followed by increasing the amount of B to 60% until 150 minutes. The

column and the trap column were flushed with 90% B for 15 minutes, until 160 minutes, which was followed by equilibration of 25 minutes for the separation column. Blank samples (injection of loading solvent) were run between sample injections for cleaning the separation system and preventing carry-over.

Before mass spectrometric detection and analysis, peptides were also identified using the UV at 214 nm in a 3nl cell. Mass spectrometric detection and MS/MS analysis was performed using the Q-Exactive Orbitrap BioPharma (ThermoFisher, Bremen, Germany). Peptides were introduced into the nano electrospray source (ESI) after the UV cell and the ionization was performed using the steel needle with 20 µm inner diameter and 10µm tip. Needle voltage was set to 2kV in positive mode and the top 10 ions were selected for MS/MS analysis (fragmentation), resolution was set to 70.000 for full MS scans, ions with single charge were excluded from MS/MS analysis and fragmented ions were excluded for 60 seconds from further fragmentation. Raw MS/MS files were converted into “mgf” by applying *MS_Convert* (http://proteowizard.sourceforge.net/tools.shtml). Database search (Sus scrofa, http://www.uniprot.org/proteomes/UP000008227, version from November 2017) was performed by submitting the “mgf” files to MASCOT v. 2.6.0 (Matrix Science, London, UK) through ProteinScape (Bruker, Bremen, Germany) and using following parameters:

- Taxonomy: Sus scrofa
- Modifications: carbamidomethyl on C as fixed, deamidation on N and Q, carboxymethylation on M and phosphorylation S and T as variable.
- Peptide tolerance was set to 20 ppm and the MS/MS tolerance to 0.05Da
- Trypsin was selected as the enzyme used and two missed cleavages were allowed
- False discovery rate (FDR) was set to 1% and the decoy database search was used for estimating the FDR.

Fichtenbaum, A., Schmid, R., & Mitulović, G. (2016). Direct injection of HILIC fractions on the reversed-phase trap column improves protein identification rates for salivary proteins. *Electrophoresis,* 37, 2922-2929.

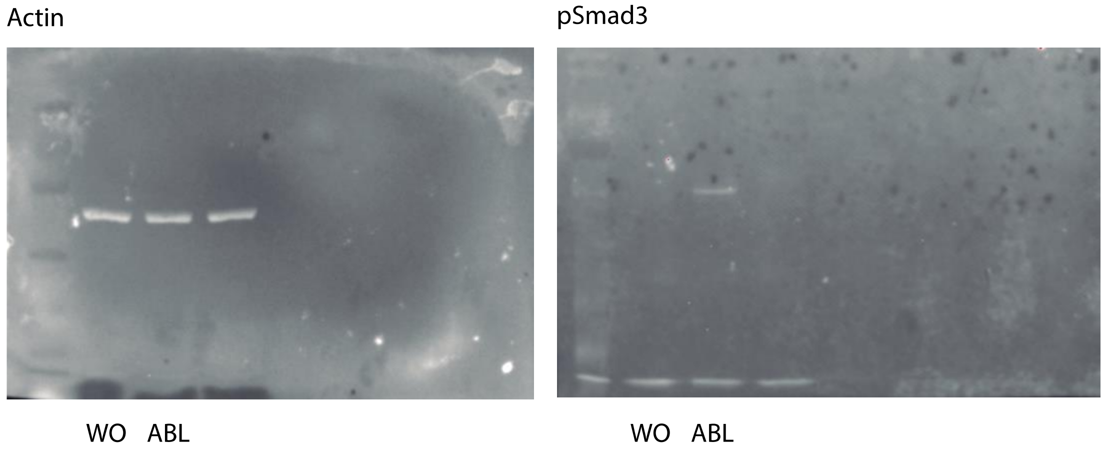

Supplementary Figure 1. Full-length blots, incubation of gingival fibroblasts with ABL caused an increased phosphorylation of Smad3.

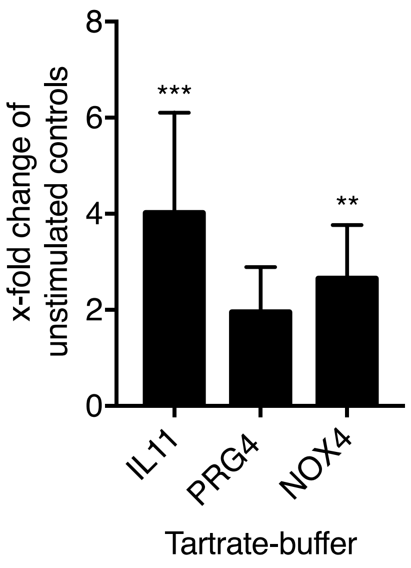

Supplementary Figure 2. ABL prepared with tartrate-buffer at pH 4.7 changes the expression of IL11 and NOX4 but not PRG4. N=3-5. Data represent the mean ± SD. ** $P < 0.01$, *** $P < 0.001$, by two-tailed Mann-Whitney test.
